# Chicago Veterinary Society

**Published:** 1900-03

**Authors:** Joseph B. Clancy

**Affiliations:** Secretary


### CHICAGO VETERINARY SOCIETY.

The regular monthly meeting of this Society was called to order on Monday evening, January 8th, in the hall, 912 Masonic Temple. President Hughes presided. Twenty-five members and several visitors were present. The new meeting place was found to be entirely satisfactory, and many expressed regret that it could not be leased for one meeting each month.

Dr. M. F. Fish's application for membership having been favorably indorsed, was presented, and his election followed. The privileges accorded to physicians by the Telephone Company were recited by Dr. E. L. Quitman, and it was resolved upon his motion that a committee be appointed to confer with the proper officials of the company and ask them to grant veterinarians the free use of drug-store telephones in calling up their offices. President Hughes named the following who are to attend to the matter: Dr. E. L. Quitman, R. G. Walker, and A. C. Worms.

Dr. W. E. Howe presented a paper on "Post-mortem Inspection of Swine," which was greeted with enthusiasm, prolonged applause, and a lively discussion, following which the Society adjourned.

THE regular monthly meeting was convened on Monday evening, February 12th. President Hughes presided, and seventeen members were present. The President announced the inability and cause for the absence of Dr. James G. Fish, who intended to read a paper on "Post-mortem Inspection of Cattle," and in its stead Dr. E. L. Quitman had kindly agreed to present an essay on "Paralysis and Treatment," which was received with much favor and ended in a extended discussion of much interest to all.

Dr. O. R. Dubia reported a case of stringhalt due to an injury received by having the foot caught in the car-track, following which a case somewhat similar was reported by Dr. E. L. Quitman, in which stringhalt was due to an injury to the vertebra. Both cases were thoroughly discussed, and the meeting adjourned.

JOSEPH B. CLANCY,
Secretary.

